# 221. Immunogenicity and Safety Study of a Quadrivalent Meningococcal Conjugate Vaccine (MenACYW-TT) when Co-administered with Routine Pediatric Vaccines in Healthy Infants and Toddlers in the US and Puerto Rico

**DOI:** 10.1093/ofid/ofae631.079

**Published:** 2025-01-29

**Authors:** James CampbellBetzana Zambrano, Mandeep S Dhingra, Sandeep Gupta, Lucia Gan, Siham Bchir, Julie Chaix, Olga Syrkina, Julie masson, Olga Lyabis, Christine Rehm

**Affiliations:** Sanofi Pasteur, Montevideo, Montevideo, Uruguay; Sanofi Pasteur, Montevideo, Montevideo, Uruguay; Sanofi, Swiftwater, Pennsylvania; Sanofi, Swiftwater, Pennsylvania; Sanofi, Swiftwater, Pennsylvania; Sanofi, Swiftwater, Pennsylvania; Sanofi, Swiftwater, Pennsylvania; Sanofi, Swiftwater, Pennsylvania; Sanofi, Swiftwater, Pennsylvania; Sanofi, Swiftwater, Pennsylvania

## Abstract

**Background:**

The incidence of invasive meningococcal disease (IMD) was 0.09 per 100,000 population in the US in 2022, with the highest rates observed in infants < 1 year of age. MenQuadfi® (MenACYW-TT) is licensed in the US against IMD for individuals 2 years and older and is under development for use from 6 weeks of age to provide protection against IMD caused by serogroups A, C, Y and W.

**Figure:**
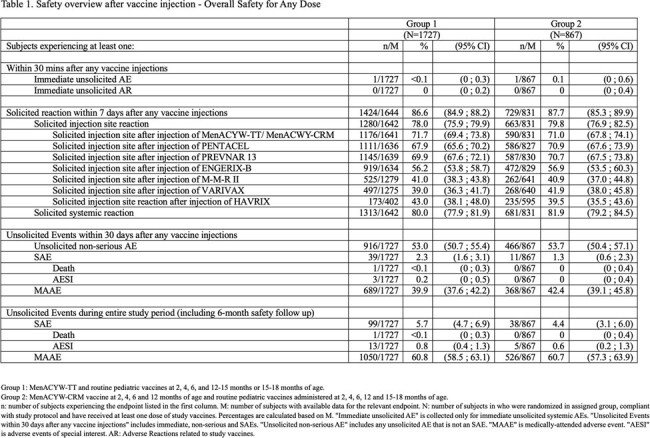

**Methods:**

In this modified double-blind, Phase-3 study (NCT03537508), 2627 healthy infants (age ≥ 42 to ≤ 89 days) were randomized 2:1 to receive 4 dose series of MenACYW-TT at 2, 4, 6, and 12-15 months or 15-18 months of age (Group 1), or 4 doses of MenACWY-CRM at 2, 4, 6 and 12 months of age (Group 2). Immunogenicity and safety of MenACYW-TT vs MenACWY-CRM were evaluated when co-administered with routine pediatric vaccines following US schedules.

**Results:**

The primary and secondary objectives were met: percentage of subjects who achieved hSBA vaccine seroresponse after the 4 dose series (at 2, 4, 6 and 12-15 months of age) was 79.4%, 97.0%, 96.4% and 97.6% for serogroups A, C, Y and W, respectively in Group 1 and was non-inferior to those in Group 2. Seroprotection rates in Group 1 were 77.9%, 99.0%, 98.3% and 98.6%, and non-inferior to Group 2 after 3 doses (at 2, 4 and 6 months of age) for the serogroups A, C, Y and W, respectively; immune responses to co-administered routine pediatric vaccines in infants and toddlers 6 weeks old to 18 months of age were non-inferior between Group 1 and 2. Overall, 2,594 (98.7%) participants had received at least one (any) dose and 2080 (79.2%) received all 4 doses of the study vaccines. Safety profile and tolerance of MenACYW-TT and MenACWY-CRM were comparable. Within 30 minutes of any vaccination, 2 participants (1 in each group) experienced a non-related immediate AE. In Group 1, 99 subjects (5.7%), and in Group 2, 38 subjects (4.4%) reported serious adverse events (SAEs) during the study; 13 subjects in Group 1 (0.8%) and 5 subjects in Group 2 (0.6%) reported an adverse event of special interest (AESI). There were 2 SAEs that lead to study discontinuation, including 1 death. All SAEs, AESIs and deaths were unrelated to the study vaccines (Table 1).

**Conclusion:**

MenACYW-TT administered with routine pediatric vaccines was immunogenic and safe in healthy infants and toddlers.

**Disclosures:**

**James Campbell**, GSK: Contracts with my University to perform vaccine clinical trials|Merck: Contracts with my University to perform vaccine clinical trials|Moderna: Contracts with my University to perform vaccine clinical trials|Pfizer: Contracts with my University to perform vaccine clinical trials|Sanofi: Grant/Research Support|Sanofi: Sanofi supported the writing of the manuscript **Betzana Zambrano, MD**, Sanofi: Employee|Sanofi: Stocks/Bonds (Public Company) **Mandeep S. Dhingra, MD**, Sanofi: Employee|Sanofi: Stocks/Bonds (Public Company) **Sandeep Gupta, MD**, Sanofi: Employee|Sanofi: Stocks/Bonds (Public Company) **Lucia Gan, PhD**, Sanofi: Employee|Sanofi: Stocks/Bonds (Public Company) **Siham Bchir, MSc**, Sanofi: Employee|Sanofi: Stocks/Bonds (Public Company) **Julie Chaix, n/a**, Sanofi: Employee **Olga Syrkina, MD**, Sanofi: Employee|Sanofi: Stocks/Bonds (Public Company) **Julie masson, n/a**, Sanofi: Contract Employee **Olga Lyabis, MD**, Sanofi: Employee|Sanofi: Stocks/Bonds (Public Company) **Christine Rehm, MD**, Sanofi: Employee|Sanofi: Stocks/Bonds (Public Company)

